# A Decade Epidemiological Study of Pediatric Burns in South West of Iran

**DOI:** 10.29252/wjps.9.1.67

**Published:** 2020-01

**Authors:** Mohammad Keshavarz, Fatemeh Javanmardi, Ali Akbar Mohammdi

**Affiliations:** 1 Student Research Committee, Shiraz University of Medical Sciences, Shiraz, Iran;; 2 Burn and Wound Healing Research Center, Shiraz University of Medical Sciences, Shiraz, Iran;; 3 Division of Plastic and Reconstructive Surgery, Department of Surgery, Shiraz University of Medical Sciences, Shiraz, Iran

**Keywords:** Pediatric, Burns, Epidemiology, Iran

## Abstract

**BACKGROUND:**

Burn is one of the most traumatic injuries and life-threatening states which expose children at a higher risk. The aim of this study was evaluating the epidemiology of pediatric burns in age less than eighteen years old during the last decade.

**METHODS:**

This cross-sectional study was carried out during 2008-2017 in Amir-al Momenin Burn Center, affiliated by Shiraz University of Medical Sciences, Shiraz, Iran. The subjects consisted of burn victims under 18 years old who were registered as outpatients and inpatients.

**RESULTS:**

During the study period, 1893 and 12431 patient were registered as inpatients and outpatients of the hospital. The burn victims were males. Children under 5 years old were prone to scald injuries more than children in any other age. More than 90% of inpatients children burned accidentally, while 116 (6.12%) burn injuries were suicidal; which was mostly seen in girls (75%, 87 out of 116).

**CONCLUSION:**

Most burns involved scalds from hot liquids especially in children age less than 5 years. Different strategies can be executed by means of broadcast flashes in mass media and educational programs through schools to show risk situation and statements calling attention to prevent childhood burn injuries.

## INTRODUCTION

Despite development in burn surgery and health care; burn trauma has remained a global concern, especially in pediatrics. Although mortality and morbidity following burn injuries have decreased in recent decades, but devasting consequences cannot be ignored in this issue.^1^ Burns in pediatrics have been found as a major health concern since they are still in growth age. So disability and impairment in aspect of psychological, social and functional are considerable subjects.^2^

Studies over the world indicated that incidence of burn injuries are in a high rate (80-90%) in developing or low-income countries.^3^^,^^4^ For instance, children in Africa burned 3 times more than world average.^5^ Based on National Burn Repository report, death in severely burn victims is a fundamental issue even in developed countries like United State America.^6^ According to literature, over half of deaths are related to fire just accounts for South-East Asia.^7^ Reports by WHO showed that over 300,000 people died by burn injury every year, also global burden of disease in 2004 was estimated 30% for patients under the age of 20 years who died due to fire-related burns.^8^^,^^9^

National Burden of Disease in Iran in 2003 demonstrated that 13^th^ most frequent cause of burden disease is burn injury is the 7^th^ for pediatrics in age 5-14 years old.^10^ There are different reasons for cause of burn in pediatrics based on their age. Over 65% of children less than 5 years old suffer from scald injuries, while fire injuries happen for 56% of cases in older age.^11^^,^^12^ Despite socioeconomic development and well-equipped burn facilities for treatment, but clear evidence demonstrated that burn incidence is preventable.^13^ Epidemiological data on pediatric burn could help to improve insight for recovery path and quality of their life and cost-effective solutions.^14^ The current study aimed to report epidemiological data in the last decade related to death, and causes of burn for inpatients and outpatients children aged under 18 years old in Fars province, Iran. 

## MATERIALS AND METHODS

This cross-sectional study was carried out during 2008-2017 in Amir-al Momenin Burn Center, affiliated by Shiraz University of Medical Sciences, Shiraz, Iran. The subjects consisted of burn victims under 18 years old who were registered as outpatients and inpatients subjects. Each patient’s information was reviewed and their demographic characteristic were obtained from the burn registry included age, sex, burn etiology, accommodation, Total Burn Surface Area (TBSA), length of stay (LOS) and final status. Data base registry was initiated by Burn and Wound Healing Research Center located in Amir-al Momenin Hospital. Data element for this database questionnaire was designed by clinicians and epidemiologists. 

## Results

During the study period, 1893 patients aged under 18 years old who were referred to burn centers and hospitalized were enrolled. The gender distribution showed male to female ratio of 1.56. The mean age for male and female patients was 6.02±5.31 and 7.20±6.09 years, respectively. The mean of LOS for all the patients was 12 days with standard deviation of 9 days. There was no significant difference between female and male patients in aspect of LOS (*p*>0.05). The TBSA ranged from 1 to 100% and it was significantly more in females than males (29.91 vs. 22.22).

The majority of these children had burns less than 50% (89.2%), while the mean age in this group was estimated 5.75±5.21 years. Higher TBSA was seen in older age, since the TBSA was estimated 44% in children 15-18 years old. It was found that the prevalence rate for mortality was 12%, with mean age of 11 years old. Of this victims, 170 (73.59%) had TBSA more than 50% (170/231). More than 90% of children burned accidentally, while 116 (6.12%) burn injuries were suicidal and mostly seen in girls (75%, 87 out of 116). 

It was found that suicidal burn victims were in older age (16±2.45). Other details for characteristics of patients were provided in Table 1. Burn etiology as the most important point about burn injuries was shown in Table 1. According to the results, scald was the most prevalent reason for burn injuries (49%), which was approximately identical in sex distribution. Also, it was the most common injury for children in age 0-4 years (74.30%). Other burn causes were seen more in males than females. 

**Table 1:** Burn etiology in relation to age and sex distribution for inpatients units

	**Scald **	**Hot objects **	**Flame**	**Explosion **	**Electrical**	**Chemical**
Total	934 (49.30%)	39 (2.10%)	604 (31.9%)	264 (13.9%)	36 (1.9%)	16 (0.8%)
Sex						
Male	573 (49.7%)	22 (1.9%)	356 (30.8%)	155 (13.4%)	33 (2.9%)	15 (1.3%)
Female	361 (48.8%)	17 (2.3%)	248 (33.6%)	109 (14.7%)	3 (0.4%)	1 (0.1%)
Age Group						
0-4 years	770 (74.3%)	24 (2.3%)	129 (12.5%)	94 (9.1%)	6 (0.6%)	13 (1.3%)
5-9 years	130 (36.5%)	6 (1.7%)	154 (43.3%)	62 (17.4%)	3 (0.8%)	1 (0.3%)
10-14 years	23 (12.8%)	5 (2.8%)	110 (61.5%)	35 (19.6 %)	4 (2.2%)	2 (1.1%)
15-18 years	11 (3.4%)	4 (1.2%)	211 (65.5%)	73 (22.7%)	23 (7.1%)	0 (0.0%)

Over the ten years period, 12431 children and adolescents aged under 18 years old attended the outpatients unit. In average, males were 5.52±5.17 years and females were 4.95±4.68 years. The gender distribution showed that the majority of patients were male (56.3%). The TBSA in outpatients ranged from 1 to 39, which was significantly more in males than girls (2.28 vs. 2.15, *p*<0.05). Accidental injuries were reported in 89.31% of cases, while suicidal and homicidal were rare and reported 0% approximately. As illustrated in Table 2, in terms of etiology; hot liquid was the most frequent cause of burns (50.40%). Other causes were hot objects (42.20%), which were equally found as the leading cause of burn in males and females; although some information was missed in outpatient unit about burn causes and age. Table 3 provides demographic characteristics of burn pediatric inpatients and outpatients. In some variables there are some missing, since our center is the main burn hospital in southwest of Iran, so the number of missing is low.

**Table 2:** Burn etiology in relation to age and sex distribution for outpatients units

	**Scald**	**Hot objects **	**Flame**	**Explosion **	**Electrical**	**Chemical**
Total	1437 (50.40%)	1202 (42.20%)	85 (3.0%)	69 (2.40)	42 (1.5%)	16 (0.6%)
Sex						
Male	746 (47.4%)	672 (42.7%)	60 (3.80%)	46 (2.9%)	38 (2.4%)	11 (0.7%)
Female	691 (54.10)	530 (41.50%)	25 (2.0%)	23 (1.8%)	4 (0.3%)	5 (0.4%)
Age Group						
0-4 years	1091 (62.6%)	586 (33.6%)	31 (1.8%)	17 (1.0%)	8 (0.5%)	11 (0.6%)
5-9 years	230 (44.40%)	235 (45.4%)	27 (5.2%)	13 (2.5%)	11 (2.1%)	2 (0.4%)
10-14 years	75 (25.7%)	170 (58.2%)	15 (5.1%)	18 (6.2%)	12 (4.1%)	2 (0.7%)
15-18 years	41 (13.8%)	211 (71.0%)	12 (4.0%)	21 (7.1%)	11 (3.7%)	1 (0.3%)

**Table 3:** Demographic characteristics of burn pediatrics

**Variable**	**Inpatients**	**Outpatients**
Age (Mean±SD)	6.48±5.66	5.27±4.97
LOS (Mean±SD)	12.72 ±9.85	-
TBSA (Mean±SD)	25.22±21.51	2.23±2.01
Sex		
Male (n, %)	(1154, 61)	(6997, 56.3)
Female (n, %)	(739, 39)	(5434, 43.7)
Final Status		
Death (n, %)	(231, 12.2)	-
Release (n, %)	(134, 7.1)	(1722, 13.80)
Transfer (n, %)	(3, 0.2)	-
Discharge (n, %)	(1519, 80.2)	-
Accommodation		
Urban (n, %)	(865, 45.7)	(314, 2.5)
Rural (n, %)	(1018, 53.8)	(10352, 83.3)
Mode of Injury		
Accidental (n, %)	(1752, 92.6)	(11103, 89.31)
Homicidal (n, %)	(11, 0.6)	(5, (0)
Suicidal (n, %)	(116, 6.12)	(2, 0)
Not specified (n, %)	(14, 0.7)	(827, 0.066)

## DISCUSSION

This epidemiological study has reported burn injury among children age 0-18 years over a 10 years peridod. According to recent analysis; it was found that males had a higher rate of injuries, which was running in parallel with other studies.^15^^,^^16^ This higher rate of incidence in males may have been tentatively explained by their percieved differences and attitudes to the risks and exposure to risk type behaviors. Also males were more daring and vibrant than females. 

The average TBSA varied from 1 to 100%, while most of studies have reported the average between 20 and 30%. However, in other countries, the averages of TBSA were less than 15% in burn children.^2^^,^^17^ In the current series; scald and flames represented the major etiological factors contributing to thermal injuries accounting for 50% of hospital references. Also, there was a clear association between age and burn type (*p*<0.05). Children under 5 years old were prone to scald injuries more than other age groups, which were similar to other studies.^5^^,^^12^^,^^18^

This may be due to children in this age that are unaware of their environment and are very curious too. Also participating in activities with high risk expose them to possible dangers without realization. Different patterns were seen in other countries (Scandinavian countries and Scotland), while burn injuries with hot liquids was followed by contact burns and burns caused by fire.^19^^,^^20^ In Canada, the second most common type of burn injury in children was fire related. The type of burn in the current study was similar to this country.^21^

In current analysis, it was revealed that rural children were found to be more vulnerable to burn injuries than urban, which were similar patterns in Ghana and France; but opposite with many other studies in other countries.^22^ Totally, the mean LOS was comparable to studies in other countries. The LOS in the current analysis was estimated 12 days. According to systematic review in the epidemiology of pediatric burns in Iran; the LOS varied from 6 to 20 days which was about the same range in other countries. Estimation of LOS for different countries was as follows: Ireland: 11 days, Republic of South Africa: 17 days, Netherlands: 11 days and South Asian countries: 27 days.^22^^-^^24^

Mortality rate in the recent analysis was estimated 12%, while the mortality rate in India was reported 13 to 29%.^25^ Due to different surgical managements, grafting and reconstructions that have been successfully used, the mortality rate, LOS and the cost of hospital care decreased in studied burn patients. As a suggestion, different strategies can be executed by means of broadcast flashes in mass media and educational programs through schools to show risk situation and statements calling attention to prevent childhood burn injuries. 

As strength point of current study, we can mention the breadth of data available for analysis during a long time data collection for inpatients and outpatients. A major limitation of this study was some missing information that the site of injuries was not recorded. In conclusion, this study indicated that adult males were the most high-risk population regarding massive burn injuries. Scalds were the main cause of pediatric burns. Furthermore, more attention should be paid to children age less than 5 years, in view of the increased risk of burn injuries. 
